# *FURIN* Promoter Methylation Predicts the Risk of Incident Diabetes: A Prospective Analysis in the Gusu Cohort

**DOI:** 10.3389/fendo.2022.873012

**Published:** 2022-03-25

**Authors:** Yan He, Yinan Li, Jianan Zhang, Linan Chen, Jing Li, Min Zhang, Qiu Zhang, Ying Lu, Jun Jiang, Xiaolong Zhang, Jianwei Hu, Yi Ding, Mingzhi Zhang, Hao Peng

**Affiliations:** ^1^Department of Epidemiology, School of Public Health, Medical College of Soochow University, Suzhou, China; ^2^Jiangsu Key Laboratory of Preventive and Translational Medicine for Geriatric Diseases, Soochow University, Suzhou, China; ^3^Department of Chronic Disease, Taicang Center for Disease Control and Prevention, Suzhou, China; ^4^Department of Central Office, Suzhou National New and Hi-Tech Industrial Development Zone Center for Disease Control and Prevention, Suzhou, China; ^5^Department of Chronic Disease, Gusu Center for Disease Control and Prevention, Suzhou, China; ^6^Department of Tuberculosis Control, Suzhou Center for Disease Control and Prevention, Suzhou, China; ^7^Department of Central Office, Maternal and Child Health Bureau of Kunshan, Suzhou, China; ^8^Department of Preventive Medicine, College of Clinical Medicine, Suzhou Vocational Health College, Suzhou, China

**Keywords:** furin, DNA methylation, diabetes, prospective observational study, Chinese

## Abstract

**Background:**

Furin has been associated with diabetes but the underlying mechanisms are unclear. As a mediator linking fixed genome and dynamic environment, DNA methylation of its coding gene *FURIN* may be involved. Here, we aimed to examine the prospective association between DNA methylation in *FURIN* promoter and incident diabetes during 4 years of follow-up in Chinese adults.

**Methods:**

DNA methylation levels in *FURIN* promoter were quantified by target bisulfite sequencing using peripheral blood from 1836 participants in the Gusu cohort who were free of diabetes at baseline. To examine the association between DNA methylation levels in *FURIN* promoter and incident diabetes, we constructed a logistic regression model adjusting for the conventional factors. Multiple testing was controlled by adjusting for the total number of CpG sites assayed using the false-discovery rate approach.

**Results:**

Among the 1836 participants free of diabetes at baseline, 109 (5.94%) participants developed diabetes during the average of 4 years of follow-up. Hypermethylation at two of the eight CpG sites assayed in the *FURIN* promoter was associated with an increased risk of diabetes, after multivariable adjustment and multiple testing correction. Every 5% increment in methylation levels at CpG1 and CpG2 were associated with a 22% (OR=1.22, 95%CI: 1.05-1.43, *P*=0.009, q=0.038) and 39% (OR=1.39, 95%CI: 1.08-1.77, *P*=0.009, q=0.038) higher risk of incident diabetes, respectively. The gene-based association analysis revealed that DNA methylation at multiple CpG loci was jointly associated with incident diabetes (*P*<0.001). Using the average methylation level of the 8 CpG loci in *FURIN* promoter revealed a similar association (OR=1.28, 95% CI: 1.02–1.62, *P*=0.037).

**Conclusions:**

These results suggested that the hypermethylation levels in *FURIN* promoter were associated with an increased risk for incident diabetes in Chinese adults.

## Introduction

Furin, a proprotein convertase that belongs to the proprotein convertase subtilisin/Kexin family (PCSK), has been suggested to participate in maintaining glucose homeostasis *via* cleavage and activation of the insulin receptor (1, 2). For example, β cell-specific *furin* knockout resulted in glucose intolerance and elevation of plasma glucose in mice (2, 3). Diabetic rats receiving retroviral vectors driving furin expression showed an amplified secretion of insulin and a significant decline in blood glucose (4). Rats receiving diminazene, a competing inhibitor of furin, have a significantly reduced level of glucose (5). The potential role of furin in glucose metabolism was also suggested by population studies. A cross-sectional study found that participants with prediabetes and diabetes had a lower level of serum furin than those with normal glucose (6). The association between serum furin and the development of diabetes was also found by a prospective cohort study (7). Further, our prior studies found that a lower level of serum furin was also associated with some metabolic dysfunctions related to diabetes, such as obesity (8), hypertension (9), and microalbuminuria (10) in Chinese adults. Moreover, genetic polymorphisms in *FURIN*, the coding gene of furin protein, have been associated with metabolic syndrome (11) and hypertension (12), both conditions shared many risk factors and mechanisms with diabetes. All these pieces of evidence suggest a potential role of furin in glucose metabolism, but the clinical translation is limited. A better understanding of the underlying molecular mechanisms would undoubtedly help the translation of furin in clinical practice for prevention and treatment of diabetes.

As a mediator linking the fixed genome with a dynamic environment, DNA methylation, the most widely studied epigenetic modification, occurred in the promoter region, in particular, could regulate gene expression and function (13, 14). We hypothesized, therefore, that *FURIN* promoter methylation may be one of the molecular mechanisms underneath the role of furin in glucose metabolism. In fact, accumulating methylation markers have been found in association with diabetes by various epigenome-wide association studies (EWAS) (15–18). Due to the high dimensional data, none of the genome-widely significant CpG loci or genes identified by prior EWAS studies were related to the *FURIN* gene. Epigenetic studies in a candidate gene would help examination of the association between *FURIN* gene methylation and diabetes. Our group has found that hypermethylation at *FURIN* promoter was associated with an increased risk of hypertension in Chinese adults in the Gusu cohort (19), but whether it is associated with the risk of diabetes is still unknown.

Based on the studies above, we hypothesized that hypermethylation in *FURIN* promoter may suppress *FURIN* gene expression, thereby was associated with the development of diabetes. Because the temporal sequence is of considerable importance for causal inferences, we aimed to examine the prospective association between DNA methylation of *FURIN* promoter and incident diabetes during an average of 4 years of follow-up in the Gusu cohort. Our study would be the first prospective study and provide initial evidence for the regulating role of *FURIN* methylation in glucose metabolism.

## Methods

### Study Participants

The Gusu cohort is a community-based prospective longitudinal study, which aims to identify new risk factors and potential interventional targets for cardiovascular disease (CVD) in middle-aged and elderly Chinese adults. The study design, survey methods, and laboratory techniques have been described previously (20). In brief, 2,706 community members aged over 30 years were recruited in 2010 at baseline and all surviving participants were invited to participate in the follow-up examination in 2014. After excluding participants who had a history of CVD at baseline (n=101), lacked blood samples (n=107), had prevalent diabetes at baseline (n=217), died during follow-up (n=23), and declined to participate in the follow-up examination (n=422), 1836 participants were included in the current analysis. All participants were free of CVD and chronic kidney disease at baseline. The protocols of the current study were approved by the Soochow University Ethics Committee. Written informed consent was obtained from all study participants.

### Quantification of *FURIN* Promoter Methylation

**Figure 1:**
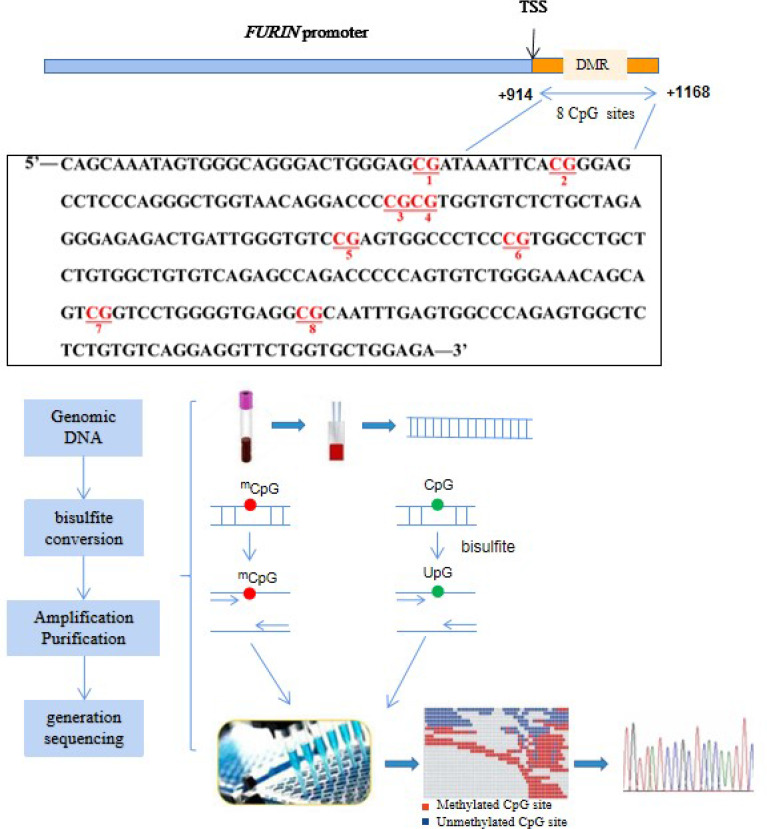
Illustration of the measurements of *FURIN* promoter methylation. The red text represents the 8 CpG loci in the *FURIN* gene promoter that were assayed in this study (+914 to +1168 bp from TSS). TSS, transcriptional start site.

DNA methylation levels in the promoter region of the *FURIN* gene were quantified by targeted bisulfite sequencing as previously described (21). Briefly, as illustrated in **Figure 1**, genomic DNA was isolated from peripheral blood mononuclear cells (PBMCs) drawn at the baseline examination. The targeted CpG sites in the *FURIN* gene were chosen based on: (a) CpG probes in the Illumina 450K array and RRBS databases; (b) regulatory elements such as promoter, enhancer, transcriptional binding sites, and (c) genomic sequences from UCSC genome browser. Based on the genomic coordinates of the *FURIN* promoter in Genome Reference Consortium Human Build 37 (GRCh37), we carefully designed the primers to detect the maximum CpG loci within the CpG islands. Following primer validation, genomic DNA was bisulfite-treated using the EZ DNA Methylation-Gold Kit (Zymo Research, Inc., CA, United States) according to the manufacturer’s protocol, which converted unmethylated cytosine into uracil and leaves methylated cytosine unchanged. The treated samples were amplified, barcoded, and sequenced by Illumina Hiseq 2000 (Illumina, Inc., CA, United States) using the paired-end sequencing protocol according to the manufacturer’s guidelines. The methylation level at each CpG dinucleotide was calculated as the percentage of the methylated alleles over the sum of methylated and unmethylated alleles. For quality control, the samples with bisulfite conversion rate <98% and the cytosine sites with average coverage less than 20× were filtered out. DNA methylation levels were finally quantified at 8 CpG loci in a region at the *FURIN* promoter. The finally targeted sequence [Chr15: 91415936-91416189, forward strand, relative to the transcription start site (TSS): +914bp to +1168bp] was also presented in **Figure 1**.

### Measurement of Fasting Glucose and Definition of Incident Diabetes

Blood samples were obtained from all participants by venipuncture in the morning after a requested overnight fast (at least 8 h). Fasting plasma glucose (FPG) was measured using an automatic biochemical analyzer (Hitachi 7020, HITACHI, Japan) at both baseline and follow-up examinations. Diabetes was defined as the presence of one of the following: (a) an FPG level of 7.0 mmol/L or higher and (b) a self-reported previous diagnosis by health care professionals and current use of either insulin or oral hypoglycemic medication (22). Incident diabetes was defined as those who were free of diabetes at baseline but developed diabetes at the follow-up examination or initiated hypoglycemic medications during follow-up.

### Measurement of Conventional Risk Factors at Baseline

Demographic data including age, sex, and education level were obtained using standard questionnaires administered by trained staff. Smoking status was defined as current smoking or not. Current smoking was defined as having smoked at least 100 cigarettes in a lifetime or as a regular and current smoker. Drinking status was defined as current drinking or not. Current drinking was defined as having consumed any type of alcoholic beverage ≥ 12 times during the past year. Body weight (kg) and height (cm) were measured by trained staff with the participants wearing light clothes and no shoes. Body mass index (BMI) was calculated by dividing the weight in kilograms by the square of height in meters (kg/m^2^). Blood pressure was consecutively measured three times using a standard mercury sphygmomanometer in a sitting position after participants rested for at least 5 minutes. The first and fifth Korotkoff sounds were recorded as systolic blood pressure (SBP) and diastolic blood pressure (DBP), respectively. The means of the three measurements were used for data analyses. Blood lipids, including total cholesterol, triglycerides, low-density lipoprotein cholesterol (LDL-C), and high-density lipoprotein cholesterol (HDL-C) were measured by standard laboratory methods using an automatic biochemical analyzer (Hitachi 7020, HITACHI, Japan).

### Statistical Analysis

All statistical analyses were conducted using R statistical software (version 4.0.2, Vienna, Austria). Baseline characteristics of study participants were presented in participants who developed diabetes and those who did not during follow-up. DNA methylation levels at each CpG site were compared between the two groups of participants using a Student’s t-test. To further examine the association between DNA methylation at each single CpG site and the risk of incident diabetes, we constructed a logistic regression model in which incident diabetes was the dependent variable and each CpG methylation level (per 5% increment) was the independent variable, adjusting for age, sex, current smoking, current drinking, BMI, LDL-C, HDL-C, and SBP at baseline. The false discovery rate (FDR) method was adopted to correct multiple testing errors by adjusting for the total number of CpG sites assayed, and an FDR-adjusted *P*-value (i.e., q value) less than 0.05 was considered statistically significant.

In addition to single CpG, the joint association of multiple CpG sites with incident diabetes was also examined. We first used the average methylation level of multiple CpG sites as a substitute for the methylation level of the targeted region and similarly examined its association with incident diabetes. Then we employed the weighted truncated product method (wTPM) (23) to test the joint association based on the single CpG associations with incident diabetes. This method combines *P*-values of all CpG sites that reach a predetermined threshold (e.g., raw *P* < 0.1 in this study). The regression coefficient of each individual CpG methylation generated from the above single CpG association analysis was included as weight in the wTPM statistic. This method has been extensively applied to epigenetic analysis (24, 25).

### Secondary Analysis

We additionally calculated continuous net reclassification index (NRI) and integrated discrimination improvement (IDI) to examine whether *FURIN* promoter methylation could improve the prediction performance over traditional risk factors (26). The improvement of predictive performance was tested by the likelihood ratio test (27). Genotype-Tissue Expression (GTEx) database was applied to examine whether *FURIN* gene was expressed in PBMCs. Integrative DNA methylation (iMethyl) database was applied to examine whether the CpG sites assayed presented in PBMCs and regulated gene expression (28).

## Results

### Baseline Characteristics of Study Participants

A total of 1,836 participants (mean age of 52.4 years, 37.09% men) who were free of diabetes at baseline and successfully followed up were included in the current study. Among them, 109 (5.94%) participants developed new diabetes during 4 years of follow-up. **Table 1** shows the baseline characteristics of study participants according to the incidence of diabetes. Compared with participants who remained free of diabetes, those who developed incident diabetes were more likely to be older and male, had higher levels of BMI, SBP, DBP, FPG, TC, and had a lower level of HDL-C at baseline (all *P*<0.05). We did not find significant differences in other listed variables.

**Table 1:** Baseline characteristics of study participants according to incident diabetes during follow-up.

Characteristics	Total	Incident diabetes		
		No	Yes	*P*
No. of participants	1836	1727	109	–
Age, years	52.4 ± 9.1	52.1 ± 9.1	56.9 ± 7.7	<0.001
Sex, males (%)	681 (37.09)	627 (36.31)	54 (49.54)	0.007
Education, high school or above (%)	342 (18.63)	318 (18.41)	24 (22.02)	0.318
Current smoking, n (%)	423 (23.04)	392 (22.70)	31 (28.44)	0.206
Current drinking, n (%)	340 (18.52)	322 (18.65)	18 (16.51)	0.668
Body mass index, kg/m^2^	24.66 ± 3.40	24.55 ± 3.22	26.38 ± 5.22	<0.001
Systolic blood pressure, mmHg	129.6 ± 16.4	129.2 ± 16.1	136.3 ± 19.1	<0.001
Diastolic blood pressure, mmHg	84.7 ± 9.2	84.6 ± 9.1	86.9 ± 10.2	0.021
Fasting glucose, mmol/L	5.11 ± 0.66	5.07 ± 0.63	5.84 ± 0.75	<0.001
Total cholesterol, mmol/L	5.18 ± 1.62	5.17 ± 1.65	5.38 ± 1.10	0.068
Triglycerides, mmol/L	1.40 ± 1.41	1.37 ± 1.06	1.87 ± 2.45	0.036
Low-density lipoprotein cholesterol, mmol/L	3.00 ± 0.76	2.99 ± 0.76	3.11 ± 0.69	0.090
High-density lipoprotein cholesterol, mmol/L	1.52 ± 0.47	1.53 ± 0.47	1.41 ± 0.37	0.002

Results are expressed with mean ± SD, unless otherwise noted. SD, Standard deviation.

### Single CpG Association Between *FURIN* Promoter Methylation and Incident Diabetes

**Figure 2:**
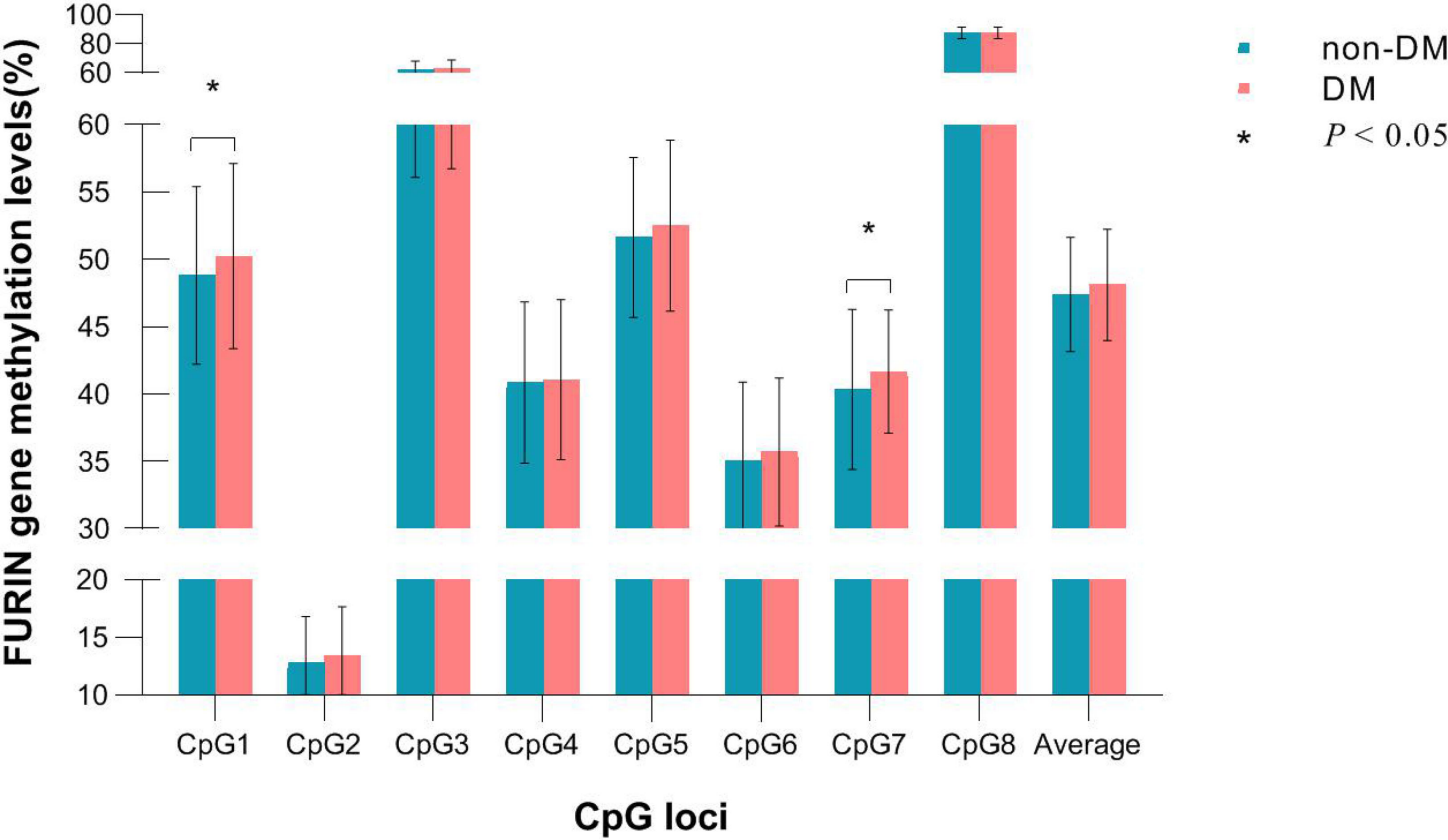
DNA methylation levels of each CpG in participants who developed incident diabetes and those who did not during follow-up.

Of the 8 CpG loci assayed, DNA methylation levels at CpG1 (50.24 ± 6.88 *vs.* 48.82 ± 6.59, *P*=0.038) and CpG7 (41.66 ± 4.56 *vs.* 40.35 ± 5.94, *P*=0.005) were significantly higher in participants who developed incident diabetes than those who did not (**Figure 2**). After adjustment for covariates, hypermethylation at CpG1 (OR=1.22, 95%CI: 1.05-1.43, raw *P*=0.009), CpG2 (OR=1.39, 95%CI: 1.08-1.77, raw *P*=0.009), and CpG7 (OR=1.23, 95%CI: 1.04-1.47, raw *P*=0.020) were nominally associated with an increased risk of incident diabetes. After further correction for multiple testing, CpG1 and CpG2 survived (all q<0.05), whereas CpG7 held a bottom-line significance with a q value of 0.052 (**Table 2**).

**Table 2:** The prospective association between baseline *FURIN* promoter methylation and incident diabetes.

CpGloci	Genomic position, GRCh37	Relative to TSS, bp	Methylation level, %	OR (95%CI) ^*^	Raw *P*	q
Single CpG association						
CpG1	Chr15:91415964	1196	48.90 ± 6.61	1.22 (1.05-1.43)	0.009	0.038
CpG2	Chr15:91415975	1207	12.83 ± 4.04	1.39 (1.08-1.77)	0.009	0.038
CpG3	Chr15:91416006	1238	62.19 ± 6.07	1.10 (0.94-1.30)	0.239	0.318
CpG4	Chr15:91416008	1240	40.86 ± 5.99	1.08 (0.92-1.28)	0.389	0.444
CpG5	Chr15:91416047	1279	51.67 ± 5.98	1.16 (0.98-1.36)	0.081	0.162
CpG6	Chr15:91416060	1292	35.11 ± 5.82	1.12 (0.95-1.33)	0.191	0.305
CpG7	Chr15:91416118	1350	40.43 ± 5.89	1.23 (1.04-1.47)	0.020	0.052
CpG8	Chr15:91416134	1366	87.50 ± 4.04	0.98 (0.77-1.26)	0.873	0.873
Gene-based association						
Average			47.43 ± 4.23	1.28 (1.02-1.62)	0.037	–
wTPM					<0.001	

^*^Risks of incident diabetes associated with per 5% increase in DNA methylation level at baseline, after adjusting for age, sex, cigarette smoking, alcohol consumption, body mass index, systolic blood pressure, low- and high-density lipoprotein cholesterol at baseline.

OR, odds ratio; 95% CI, 95% Confidence interval; wTPM, weighted truncated product method.

### Gene-Based Association Between *FURIN* Promoter Methylation and Incident Diabetes

The average methylation level of the 8 CpG loci (48.12 ± 4.13 *vs.* 47.39 ± 4.24, *P*=0.079) was slightly higher in participants who developed incident diabetes than those who did not, but not significantly. (**Figure 2**). It was significantly associated with a higher risk of incident diabetes (OR=1.28, 95% CI: 1.02–1.62, raw *P*=0.037), after adjusting for conventional risk factors (**Table 2**). The wTPM consistently revealed that DNA methylation of the 8 CpG loci at the *FURIN* promoter as a whole was significantly associated with incident diabetes (*P*<0.001).

### Results of Secondary Analysis

We further examined whether DNA methylation levels at CpG1, CpG2, CpG7, and the average methylation level in the targeted region of the *FURIN* gene could improve the prediction performance for the risk of diabetes. As shown in **Table 3**, adding DNA methylation at CpG1 (NRI=0.171, *P* = 0.009), CpG2 (NRI=0.270, *P *= 0.009), CpG7 (NRI=0.217, *P *= 0.018), and the average methylation level (NRI=0.210, *P *= 0.037) significantly improved the discriminatory ability for diabetes over conventional risk factor, although none of the IDI reached a statistical significance. Genotype-Tissue Expression (GTEx) showed that *FURIN* gene was also expressed in PBMCs. Integrative DNA methylation (iMethyl) database showed that DNA methylation at 6 of the 8 CpG sites assayed could occur in PBMCs and five of them were eQTMs, i.e., were associated with the expression levels of *FURIN* gene (28).

**Table 3:** Reclassification and discrimination statistics for diabetes risk prediction by *FURIN* promoter methylation.

Predictive models	NRI		IDI	
	95%CI	*P*	95%CI	*P*
Conventional model	reference		reference	
Conventional model + CpG1 methylation	0.171 (-0.022-0.364)	0.009	0.004 (-0.001-0.009)	0.078
Conventional model + CpG2 methylation	0.270 (0.079-0.461)	0.009	0.003 (-0.002-0.008)	0.199
Conventional model + CpG7 methylation	0.217 (0.025-0.409)	0.018	0.003 (-0.000-0.007)	0.061
Conventional model + average methylation	0.210 (0.018 - 0.402)	0.037	0.002 (-0.001 - 0.005)	0.324

NRI, net reclassification improvement; IDI, integrated discrimination index.

Conventional model included age, sex, cigarette smoking, alcohol consumption, body mass index, systolic blood pressure, low- and high-density lipoprotein cholesterol at baseline.

## Discussion

In the prospective cohort study of Chinese adults in the Gusu cohort, we demonstrated for the first time that *FURIN* promoter hypermethylation at baseline was significantly associated with an increased risk of future diabetes, independent of conventional risk factors. DNA methylation at two CpG sites located at Chr15:91415975 and Chr15:91416118 could improve the prediction performance for the risk of diabetes over conventional risk factors. *FURIN* promoter methylation may participate in the development of diabetes beyond lifestyles and metabolic factors. These findings suggest that *FURIN* promoter hypermethylation might serve as a potential predictor and a probable therapeutic target for diabetes.

Although no direct evidence, previous findings from other dimensions supported the association between *FURIN* promoter hypermethylation and diabetes identified by our study. For instance, animal experiments suggested that furin is essential for pancreatic β-cell function, and dysregulation of its activity caused β-cell dysfunction by the induction of the stress factor ATF4 in a mTORC1-dependent manner (2). Furin is also crucial for the acidification of secretory granules in mouse pancreatic β-cells (29). βcell-specific Furin knockout (βFurKO) mice showed a significant reduction in the functional β-cells, which might be below the threshold required to maintain adequate glucose homeostasis and could directly lead to impaired pulsatile insulin secretion. It was also shown that total insulin content was strongly decreased in βFurKO cells (2, 3). In humans, the genetic polymorphisms in the *FURIN* gene were demonstrated to be significantly associated with some diabetes-related phenotypes, such as metabolic syndrome and hypertension. A case-control study conducted in Japan showed that minor A allele of rs17514846 of the *FURIN* gene was significantly associated with a decrease in TG and an increase in HDL (11). Another case-control study in Xinjiang Kazakh and Uygur populations of Chinese ethnic minority groups demonstrated that rs2071410 in the *FURIN* gene was significantly associated with hypertension and the G allele of rs2071410 may be a modest risk factor for hypertension (30). A recent study including 4678 participants from Malmö Diet and Cancer study identified a positive association between plasma furin levels and glucose, insulin, LDL-C, and BMI, as well as increased incidence of diabetes and mortality (7). Our previous cross-sectional study also found serum furin was associated with prediabetes and diabetes in Chinese adults (6). Furthermore, the relationship between elevated circulating furin levels and hypertension (9), obesity (8, 31), diabetic cardiovascular disease (32) were found in other epidemiological studies. However, the underlying molecular mechanisms behind these relationships are not yet clear.

DNA methylation is an epigenetic modification that can regulate gene expression and cause differences in disease susceptibility between individuals (33). *FURIN* promoter methylation may be a potential molecular modification that regulates furin expression underneath the relationship between furin and diabetes. Therefore, we examined the association between *FURIN* promoter methylation and incident diabetes. Our results showed that hypermethylation of the CpG sites at the *FURIN* promoter could predict a higher risk of diabetes during an average 4-year follow-up in Chinese adults (OR=1.22, 95%CI: 1.05-1.43, for CpG1 and OR=1.39, 95%CI: 1.08-1.77, for CpG2). In line with our study, the identified association between DNA methylation and diabetes has also been suggested by other studies. For example, Epigenome-wide association studies (EWASs) have identified several DNA methylation markers associated with diabetes. A nested case-control study including 25,372 individuals identified five loci that were associated with future type 2 diabetes incidence, including ABCG1, PHOSPHO1, SOCS3, SREBF1, and TXNIP (34). Another case-control study, which excluded confounding effects of anti-diabetic drugs or insulin treatment, found that the methylation promoter of the TCF7L2, the gene with the strongest effect for type 2 diabetes, was significantly different between patients with type 2 diabetes and controls and associated with fasting blood glucose levels (35). A genome-wide DNA methylation analysis of human pancreatic islets from type 2 diabetes identified 1,649 CpG sites and 853 genes with differential DNA methylation, but none was related to the *FURIN* gene (16). Leveraging an unselected population in the Gusu cohort, the prospective association between *FURIN* promoter methylation and incident hypertension was examined (36). Our study is the first epidemiological study on *FURIN* promoter methylation concerning the risk of future diabetes and provides initial evidence for the potential role of *FURIN* promoter methylation in the pathogenesis of diabetes.

Our results demonstrated that although methylation level at a single locus (CpG1 or CpG2) shows a significant association with diabetes, but it only explained a very small proportion of the risks of diabetes (<1%). Our previous study reported that methylation of the eight CpG sites in the *FURIN* promoter was highly correlated (36). Whether the joint effect of multiple CpG sites was larger? Therefore, we assumed that multiple CpG sites would exert their effects jointly or interactively in the pathogenesis of diabetes. We tested and found a significant joint association of multiple CpG methylation sites in *FURIN* promoter with incident diabetes (*P*<0.001), using a gene-bases association analysis approach. Our results may suggest that a gene-based approach simultaneously modeling the joint effect of multiple CpG sites within a gene may serve as an important method to identify the joint effect of multiple epigenetic variants on human complex phenotypes, such as diabetes, hypertension (36) and obesity (37).

To the best of our knowledge, our study represented the first to investigate the association between DNA methylation in *FURIN* promoter and diabetes in Chinese adults. The strengths of this study include careful and systemic analyses of the association between *FURIN* promoter methylation and incident diabetes in Chinese adults, comprehensive adjustments of many conventional risk factors including lifestyles and metabolic factors, and the application of a gene-based analytical approach testing the combined effect of multiple CpG-methylation sites in *FURIN* promoter on diabetes incidence. However, our study has some limitations that deserve clarification. First, DNA methylation is tissue- and cell-type specific (38). It is unclear whether or to what extent our results could reflect DNA methylation changes in important organs in glucose metabolisms, such as the pancreas and muscles. Second, although we have controlled many potential confounders, we cannot rule out the possibility of residual confounding by other unknown or unmeasured factors. Third, DNA methylation is highly variable between peoples and groups (39), the generalizability of our findings to other age groups or populations is uncertain. Therefore, the association between DNA methylation levels in *FURIN* promoter and diabetes needs to be further investigated and explored in multi-ethnic studies and large sample populations.

In conclusion, our study demonstrated that hypermethylation in *FURIN* promoter at baseline could predict an increased risk of future diabetes in Chinese adults. It indicated that *FURIN* promoter methylation could serve as a predictor for the identification of individuals at high risk for diabetes during primary prevention, but more evidence is needed to establish the causality between *FURIN* promoter methylation and diabetes.

## Data Availability Statement

The data presented in the study are deposited in the Dryad repository, accession number doi: 10.5061/dryad.7m0cfxpwn.

## Ethics Statement

The studies involving human participants were reviewed and approved by the Soochow University Ethics Committee. The patients/participants provided their written informed consent to participate in this study.

## Author Contributions

YH, YLi, and JZ performed the statistical data analysis and drafted the manuscript. HP and MZZ developed the concept of the study design and contributed to drafting the manuscript. LC, JL, MZ, QZ, YLu, JJ, XZ, JH, and YD obtained the clinical data and critically reviewed the manuscript. JJ, XZ, and JH contributed to the interpretation of the results. All authors contributed to drafting the final versions of the manuscript. All authors have read and approved the final manuscript.

## Funding

This study was supported by the National Natural Science Foundation of China (NO. 82173596, 81903384, and 81872690), the Suzhou Municipal Science and Technology Bureau (NO. SYS2020091 and SKJY2021040), the Youth Program of Science and Technology for Invigorating Health through Science and Education in Suzhou (NO. KJXW2020084, KJXW2019067, and KJXW2018078), Suzhou Key Technologies of Prevention and Control of Major Diseases and Infectious Diseases (NO. GWZX201803 and GWZX202001), the Maternal and Child Health Project of Jiangsu Province (NO. F201721), Scientific Research Project of Jiangsu Health Committee (NO. M2020051), Natural Science Research Projects of Colleges and Universities in Jiangsu Province (NO. 20KJB330004), Key Natural Science Projects of Suzhou Vocational Health College (NO. SZWZY202002), and a Project of the Priority Academic Program Development of Jiangsu Higher Education Institutions.

## Conflict of Interest

The authors declare that the research was conducted in the absence of any commercial or financial relationships that could be construed as a potential conflict of interest.

## Publisher’s Note

All claims expressed in this article are solely those of the authors and do not necessarily represent those of their affiliated organizations, or those of the publisher, the editors and the reviewers. Any product that may be evaluated in this article, or claim that may be made by its manufacturer, is not guaranteed or endorsed by the publisher.
